# Comparison of the therapeutic effects of medication therapy, specific immunotherapy and anti-IgE (Omalizumab) in patients with hay fever

**DOI:** 10.3389/fimmu.2024.1363034

**Published:** 2024-02-28

**Authors:** Rui Tang, Xiaohong Lyu, Yibo Hou, Yongshi Yang, Guodong Fu, Liping Zhu, Lu Xue, Hong Li, Ruiqi Wang

**Affiliations:** ^1^ Department of Allergy, Beijing Key Laboratory of Precision Medicine for Diagnosis and Treatment of Allergic Diseases, National Clinical Research Center for Dermatologic and Immunologic Diseases, Peking Union Medical College Hospital, Chinese Academy of Medical Sciences, Peking Union Medical College, Beijing, China; ^2^ Department of Breast Surgery, Peking Union Medical College Hospital, Chinese Academy of Medical Sciences, Peking Union Medical College, Beijing, China

**Keywords:** hay fever, Omalizumab, subcutaneous immunotherapy, allergy, medication

## Abstract

**Background:**

Hay fever, characterized by seasonal allergic reactions, poses a significant health challenge. Existing therapies encompass standard drug regimens, biological agents, and specific immunotherapy. This study aims to assess and compare the effectiveness of anti-IgE (omalizumab), medication therapy, and subcutaneous immunotherapy (SCIT) for hay fever.

**Methods:**

Conducted as a retrospective cohort study, this research involved 98 outpatient hay fever patients who underwent routine medication, omalizumab treatment, or SCIT before the onset of the spring pollen season. A follow-up was performed one month after the start of the pollen season. The comprehensive symptoms and drug scores were used to evaluate patients with different intervention methods, facilitating a comparative analysis of therapeutic outcomes.

**Results:**

Compared with before treatment, the symptoms of patients treated with the three methods were all significantly relieved, and the medication score were significantly reduced. Patients treated with omalizumab demonstrated higher symptoms and medication scores than SCIT group before treatment, but similar scores after treatment, which were both lower than medicine treatment group. After treatment with omalizumab or SCIT, patients in both groups had significantly lower medication scores than the medication group and were close to no longer using medication for symptom relief. The mountain juniper-sIgE was significantly higher after treatment than before treatment in both medicine treatment group and omalizumab treatment group.

**Conclusion:**

Omalizumab and SCIT offer superior effects than medication therapy in hay fever patients.

## 1 Introduction

Hay fever is a disease with IgE mediated allergic rhinitis, allergic conjunctivitis, asthma, urticaria, allergic dermatitis and other symptoms in patients with specific constitution after inhalation or exposure to pollen allergens (1). The occurrence of symptoms is related to the region and dissemination season of pollen. When inhaled allergens contact the eyelids, ocular conjunctiva and nasal mucosa, they will cause eyelid swelling, conjunctival congestion, aqueous secretions, nasal itching, runny nose, sneezing, nasal congestion and other symptoms (1, 2). These symptoms seriously affect the quality of life of patients, as well as the learning efficiency of adolescent patients (3). In the four seasons of the year, pollinosis is mainly caused by outdoor pollen transmitted by wind, including tree pollen, grass pollen and weed pollen (4). The spring pollen that causes hay fever in Beijing is mainly juniper, and the fall pollen is mainly Artemisia absinthium and Humulus lupulus (5–7).

The current treatment of hay fever patients mainly aims at controlling symptoms and depends on many factors, such as the severity of symptoms and the self-management of patients. The treatment of hay fever mainly lies in the control of symptoms, rather than the quality of life (8). The symptoms of allergic rhinoconjunctivitis seriously affect the quality of life of patients. Anti-histamine medications, local steroid medications, and mast cell stabilizers are currently the main medicine treatments utilized to reduce the symptoms. More research has been devoted to the use of allergen specific immunotherapy for the treatment and prevention of hay fever. Allergens can be used subcutaneously or sublingually (9). In recent years, the widespread use of biological agents has played an important role in the treatment of allergic diseases. Omalizumab, as a typical representative, has been approved by the FDA for the treatment of allergic asthma and chronic urticaria (10, 11). Omalizumab is a monoclonal antibody against IgE. Omalizumab has been proved to have the advantages of high safety and low side effects in the treatment of allergic diseases (12), and may be effective for patients who are ineffective in traditional medicine treatment. Omalizumab has been approved in Japan for the treatment of severe hay fever (11).

In this study, we retrospectively compared the efficacy of different treatments for hay fever, including standard medicine therapy, omalizumab, and subcutaneous allergen specific immunotherapy (SCIT).

## 2 Materials and methods

### 2.1 Study design and patients

The study was approved by the ethics committee of Peking Union Medical College Hospital (No. K4858), and written informed consent was obtained from each patient before participation.

This was a retrospective cohort study. This study included a total of 98 outpatient patients diagnosed as hay fever who received traditional standard medicine treatment, omalizumab treatment, and SCIT treatment. The interval of the study was from March 1 to April 30, 2023, the whole spring mountain juniper pollen season. All patients were followed up after the start of the pollen season.

Omalizumab or SCIT were used alone without standard medicine treatment. The dose of Omalizumab was 150 mg under 18 years of age and 300 mg over 18 years of age. Patients who received SCIT have undergone regular desensitization treatment according to the allergy department plan of Peking Union Medical College Hospital for one year or more. Patients with different intervention methods were evaluated through comprehensive symptoms and medication scores to compare the therapeutic effects of traditional medicine, omalizumab, and SCIT on hay fever. 

### 2.2 Diagnosis and inclusion criteria

The diagnosis of hay fever is based on the Chinese Guidelines for the diagnosis and treatment of allergic rhinoconjunctivitis (2022, Revised Edition). Patients should meet the following three criteria in order to obtain a confirmed diagnosis of allergic rhinoconjunctivitis: 1) Symptoms: ≥ 2 symptoms: paroxysmal sneezing, nasal mucus, itching, and nasal congestion, lasting for more than 1 hour per day, which may be accompanied by tears, itchy eyes, and red eyes; 2) Body sighs: pale or swollen nasal mucosa, watery nasal secretions; 3) Allergen testing: At least one type of pollen tested positive and/or serologically specific IgE test result was positive in the skin puncture (10). Specific IgE levels were detected through enzyme-linked immunosorbent assay (ImmunoCAP system).

### 2.3 Assessment of efficacy

Hay fever visual analogue scale (VAS) score, symptom score and drug score were used to understand the efficacy of different intervention methods. The hay fever VAS score was evaluated based on the severity of nasal symptoms (0: no symptoms, 10: very severe symptoms). Symptom scores are based on nasal and ocular conjunctival symptoms including itchy nose, sneezing, runny nose and nasal congestion, and itchy eyes. (1: mild symptoms, 2: moderate symptoms, 3: severe symptoms, the maximum score is 3 points, i.e. 15 points/divided by 5 symptoms). Medication scores were based on oral and/or topical (eye or nose) non sedative H1 antihistamines (H1a), intranasal corticosteroids (INS) with/without H1a, and oral corticosteroids with/without INS, with/without H1a, with a maximum score of 24 (13, 14). Higher medication scores represent more medications that need to be used to control symptoms. The medication and symptom scores were the summation of the above two scores. Patients in the three treatment groups had symptom scores and medication scores done daily after the start of treatment, and the average of these scores was taken for comparison.

### 2.4 Statistical analyses

Shapiro Wilk test tested the normality of continuous data. Normal variables were presented as (mean ± standard deviation, SD) and analyzed by *t*-test or ANOVA analysis. *P* values for two-by-two comparisons in the ANOVA analysis have been statistically adjusted using Tukey’s multiple comparisons test. *P* values were considered statistically significant when <0.05. The data were statistically analyzed in prism 9.0 (Graphpad Software, Inc).

## 3 Results

### 3.1 Participants’ characteristics

A total of 98 patients were included in this study, of which 31 received standard medicine treatment, 32 received omalizumab treatment, and 35 received SCIT for one year or more. A questionnaire survey has been conducted to evaluate the control of symptoms related to hay fever. All patients were divided into three groups based on different intervention methods, and there were no significant differences in gender among the three groups (**Table 1**). The age of patients with omalizumab was slightly younger than that of SCIT patients (34.5 ± 11.62 vs 41.33 ± 8.88, adjusted *P* = 0.024). SCIT patients had a slightly longer duration of illness than patients in the medication treatment group (7.69 ± 4.09 vs 5.00 ± 3.10 months, adjusted *P* =0.014).

**Table 1:** Demographic and Characteristics of the hay fever patients.

Characteristic	Medication treatment (n=31)	Omalizumab (n=32)	SCIT (n=35)	*P* value
**Age, mean ± SD**	37.42 ± 10.95	34.5 ± 11.62	41.33 ± 8.88	0.030
**Female (%)**	20(64.52%)	15(46.88%)	21(60%)	0.343
**Duration of disease, mean ± SD, months**	5.00 ± 3.10	5.88 ± 3.44	7.69 ± 4.09	0.013

### 3.2 Symptom scores and the number of particles in the air

**Figure 1:**
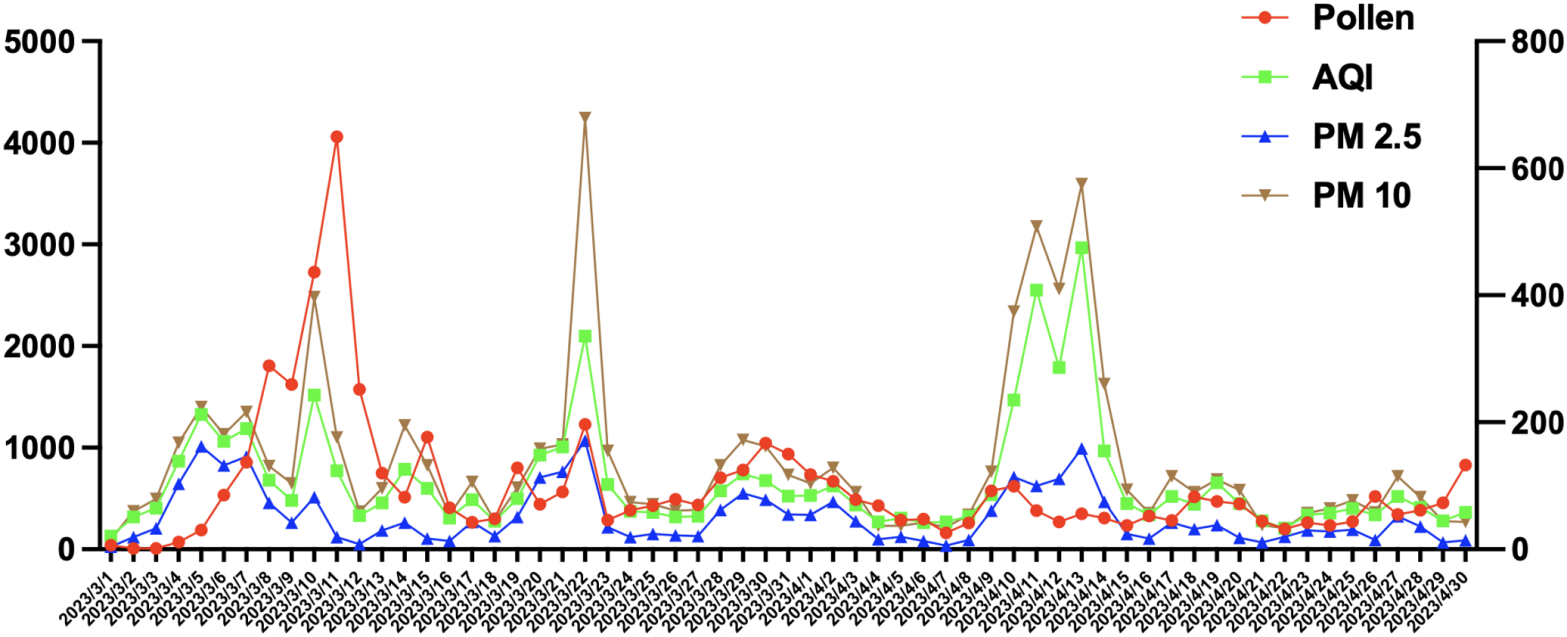
The symptom scores of patients with hay fever were consistent with the number of particles in the air. Particles index included Pollen count, AQI, PM2.5 and PM 10.

Data on pollen count, PM2.5, PM10, and air index in Beijing area in March and April were collected. The symptom scores of patients with hay fever were consistent with the number of particles in the air (**Figure 1**).

### 3.3 Comparison of pre- and post-treatment efficacy of the three treatments

**Figure 2:**
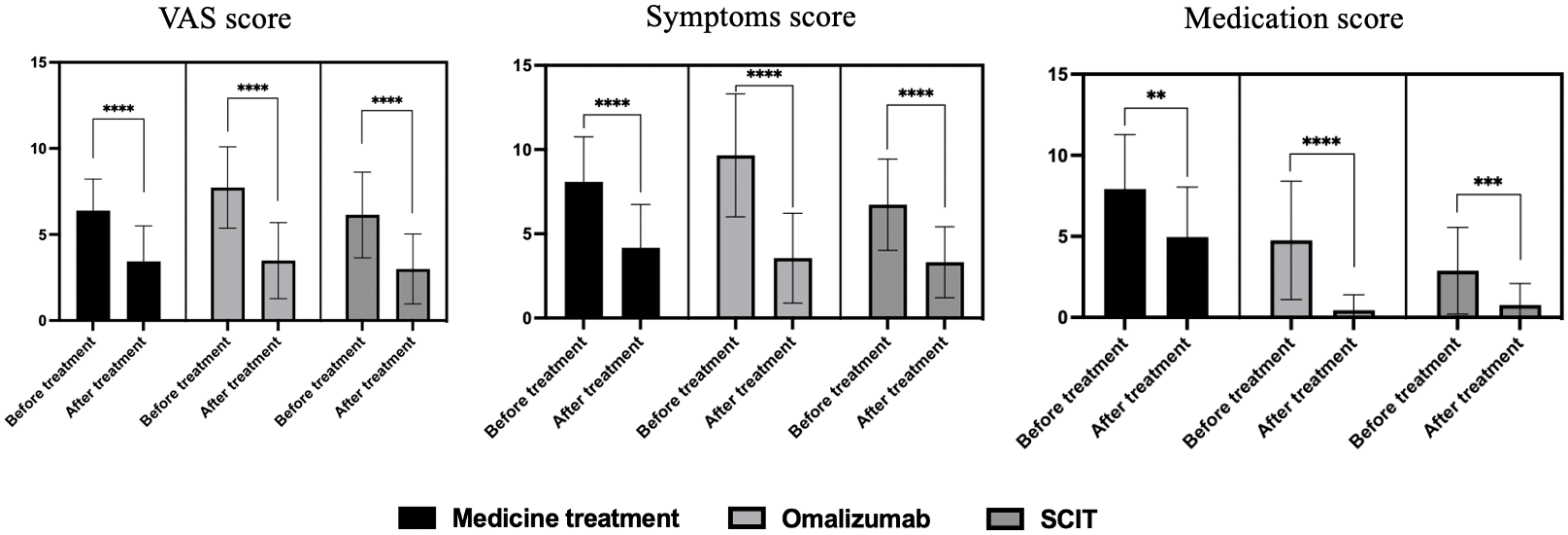
VAS score, symptom score, and medication score before and after treatment of three therapies on hay fever patients. **P<0.01, ***P<0.001, ****P<0.0001.

We evaluated the impact of three treatment methods on hay fever mainly through VAS score, symptom score, and medication score (**Figure 2**). Patients showed significant decreases in VAS scores, symptom scores, and medication scores with medication, omalizumaband SCIT therapy. After the arrival of the pollen season, the medication scores of patients in the omalizumab group and patients in the SCIT group were significantly lower than before, almost close to zero, after using the respective treatments.Yet patients in the medication group still had medication scores close to 5, albeit down from before standardized treatment.

### 3.4 Cross-sectional comparison of the efficacy of three treatments

**Figure 3:**
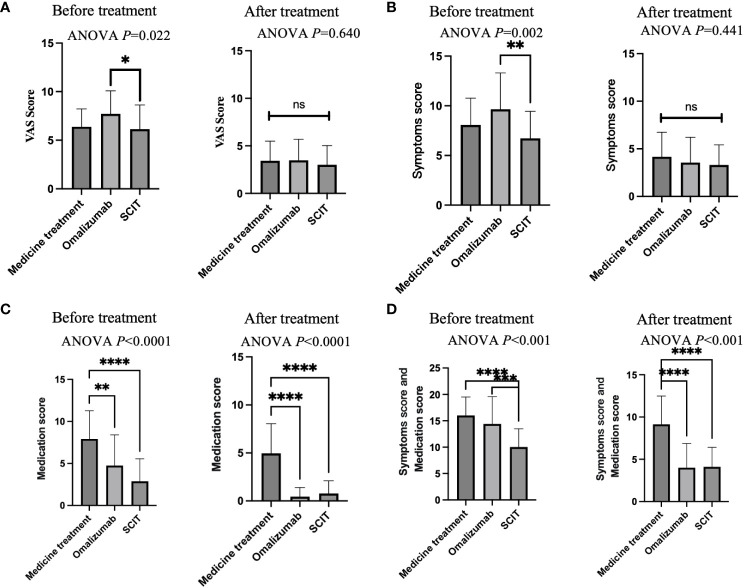
A cross-sectional comparison of the efficacy of three treatments. **(A)** VAS score. **(B)** Symptoms score. **(C)** Medication score. **(D)** Symptoms score and medication score. Notes: ANOVA P-values represent between-group differences in ANOVA comparisons. Two-by-two comparisons are indicated by asterisks. * adjusted *P*<0.05, ** adjusted *P*<0.01, *** adjusted *P*<0.001, **** adjusted *P*<0.0001. ns, Not Statistically.

Separate side-by-side comparisons were made between pre- and post-treatment scores of patients using the three therapies. Before treatment, omalizumab patients had the highest VAS scores, significantly higher than those treated with SCIT (**Figure 3A**). After treatment, there was no difference in VAS scores among patients on the three therapies. Before treatment, patients on omalizumab had significantly higher symptom scores than SCIT patients (**Figure 3B**). There was no difference in symptom scores among the three therapies at post-treatment. Before treatment, medication-treatment patients had significantly higher medication scores than omalizumab-treated and SCIT-treated patients (**Figure 3C**). After treatment, medication scores remained higher in medicated patients than in omalizumab and SCIT patients. Prior to treatment, the sum of symptom and medication scores was significantly lower in SCIT patients than in the other two groups (**Figure 3D**). At post-treatment, patients in the medication group had significantly higher symptom and medication scores than the other two groups.

### 3.5 Total-IgE and Mountain juniper-sIgE level before and after treatment

**Figure 4:**
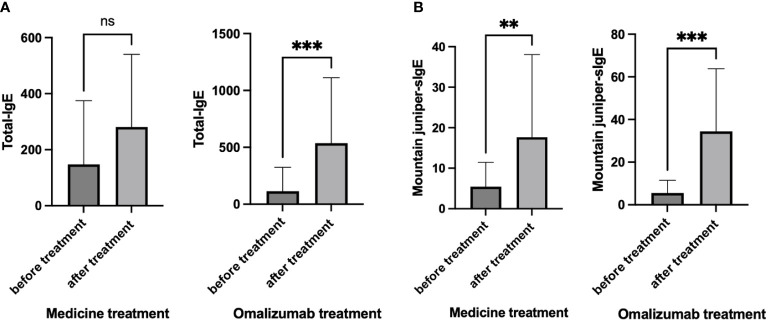
**(A)** Total-IgE and **(B)** Mountain juniper-sIgE before and after treatment in the medicine treatment group and the omalizumab group. ** *P*<0.01, *** *P*<0.001. ns, Not Statistically.

We examined the levels of total-IgE and mountain juniper-sIgE before and after treatment in the medicine treatment group and the omalizumab group (**Figure 4**). It was found that there was no significant change in total-IgE, but the level of mountain juniper-sIgE was significantly higher in this group of patients after medicine treatment. After omalizumab treatment, there was a significant increase in total-IgE and mountain juniper-sIgE levels in this group of patients.

## 4 Discussion

In this retrospective cohort study, we systematically evaluated and compared the efficacy of three hay fever treatments: medication, omalizumab, and SCIT. Utilizing patient-controlled data, our findings demonstrate that all three treatments—medication, omalizumab, and SCIT—effectively reduced VAS scores,symptom scores and medication scores among hay fever patients. Notably, the omalizumab-treated and SCIT-treated group exhibited a reduced reliance on medications. Upon intergroup analysis, our study revealed a predilection for omalizumab treatment in patients with higher pre-treatment VAS and symptom scores. However, post-treatment VAS scores and symptom scores did not exhibit significant differences among the three treatment groups. This suggests that the superior efficacy of omalizumab, particularly in addressing hay fever symptoms, might render it a more favorable option compared to medication and SCIT.

Furthermore, our investigation identified that both omalizumab and SCIT resulted in lower post-treatment symptom and medication scores when contrasted with the medication-only group (**Figure 3D**). This underscores the potential advantages of omalizumab and SCIT over conventional medication in achieving improved therapeutic outcomes for hay fever patients. In summary, our study contributes valuable insights into the comparative effectiveness of hay fever treatments, highlighting the potential superiority of omalizumab and SCIT over traditional medication. These findings have significant implications for clinical decision-making and the pursuit of more tailored and efficacious therapeutic approaches for hay fever management.

Corticosteroids, antihistamines, and leukotriene receptor antagonists are established as first-line treatments for hay fever due to their efficacy in alleviating nasal and conjunctival symptoms (15). Omalizumab, an IgE-specific antibody, has gained approval for managing moderate to severe asthma and chronic urticaria (10). Extensive clinical trials in the United States and Europe over several years have demonstrated promising outcomes in treating allergic rhinitis, showing efficacy even in cases of single resistance (16, 17). A systematic review and meta-analysis confirm the favorable efficacy and safety profile of omalizumab for patients with allergic rhinitis inadequately controlled by conventional therapies (17). Building upon our previous research, we found that prophylactic omalizumab injections before the pollen season surpass conventional drug therapy in alleviating symptoms and enhancing overall quality of life (12).

Allergen immunotherapy, with a history spanning over a century, has established both short-term and long-term efficacy. Existing research demonstrates its ability to alleviate allergic symptoms, decrease drug usage, enhance quality of life even post-treatment cessation, and hinder the progression of allergic conditions such as hay fever, allergic rhinitis, and asthma, while preventing new sensitizations (18). Christian Woehlk et al.’s study indicates that allergen immunotherapy effectively mitigates exacerbations in seasonal and perennial allergic asthma, as well as the risk of lower respiratory tract infections (9). Recent research, focusing on improving subcutaneous immunotherapy products for grass pollen allergic rhinitis, has yielded positive short-term outcomes, notably reducing patient symptoms and comprehensive drug scores (16). Our findings further affirm that subcutaneous immunotherapy effectively manages hay fever symptoms, diminishes medication dependency, and enhances overall quality of life.

In this study, the rise in total IgE immediately after the initiation of treatment might be attributed to the short human serum half-life of IgE, which is 2.4 days, in contrast to the extended half-life of IgE bound to omalizumab complexes, which is 20 days. Furthermore, as omalizumab is an IgG class antibody with a prolonged serum half-life of 26 days, the half-life of the IgE-omalizumab complex is extended upon binding with omalizumab. Since conventional total serum IgE measurement methods, such as ImmunoCAP, cannot differentiate between IgE simplexes and complexes, total serum IgE levels may appear elevated. The serum IgE level experiences an increase for approximately 1-2 months before reaching a plateau (19). In our study, regular treatment significantly reduced patients’ symptoms, even in cases where levels of total IgE and specific IgE increased. This suggests that the severity of symptoms may not directly correlate with IgE levels. The measurement of serum total IgE and specific IgE is a widely employed *in vitro* method for allergen detection and a crucial aspect of allergic disease diagnosis (20). Elevated total IgE alone does not serve as a definitive diagnostic marker for allergic diseases; for instance, a threshold of 1000 kU/L is a key criterion in diagnosing variant pulmonary aspergillosis, underscoring its diagnostic significance (21). Specific IgE provides information on sensitization to inhaled allergens and can signal the risk of hay fever or asthma, but research indicates that asthma severity lacks a significant correlation with sIgE levels, consistent with our study results (22, 23).

In conclusion, our study firstly compared the efficacy of three interventions for hay fever patients, revealing that both omalizumab treatment and subcutaneous immunotherapy outperformed conventional drug treatment. This provides a novel reference for patient management. Omalizumab can be considered for individuals with severe symptoms who face challenges in adhering to desensitization treatment or maintaining regular long-term drug use.

## Data availability statement

The raw data supporting the conclusions of this article will be made available by the authors, without undue reservation.

## Ethics statement

The studies involving humans were approved by the ethics committee of Peking Union Medical College Hospital (No. K4858). The studies were conducted in accordance with the local legislation and institutional requirements. Written informed consent for participation in this study was provided by the participants’ legal guardians/next of kin.

## Author contributions

RT: Conceptualization, Supervision, Writing – review & editing. XL: Formal Analysis, Writing – original draft. YH: Formal Analysis, Writing – original draft. YY: Data curation, Investigation, Writing – review & editing. GF: Data curation, Writing – review & editing. LZ: Data curation, Methodology, Writing – review & editing. LX: Data curation, Methodology, Writing – review & editing. HL: Conceptualization, Funding acquisition, Supervision, Writing – review & editing. RW: Conceptualization, Writing – review & editing.
